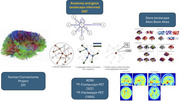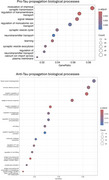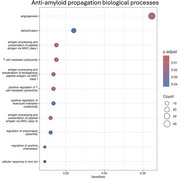# Network models suggest constitutional expression of innate immune molecules are protective against amyloid and tau propagation

**DOI:** 10.1002/alz70855_098143

**Published:** 2025-12-23

**Authors:** Fangda Leng, Yang Zhiyuan, Paul Edison, Zhaoxia Wang, ADNI

**Affiliations:** ^1^ University of California, San Francisco, San Francisco, CA, USA; ^2^ Peking University First Hospital, Beijing, Beijing, China; ^3^ University College London, London, London, United Kingdom; ^4^ Division of Neurology, Department of Brain Sciences, Imperial College London, United Kingdom, London, London, United Kingdom; ^5^ Peking University, Beijing, Beijing, China

## Abstract

**Background:**

Accumulating evidence demonstrated the trans‐regional propagation behaviour of Alzheimer's pathology across brain. Spatial patterns of amyloid and Tau pathology emergence have also been established. However, the mechanism underlying the stereotypical spatial propagation of Alzheimer's pathologies remains elusive. The current study aims to unveil molecular basis of amyloid and tau propagation network by integrating transcriptomic data with molecular imaging information via graph network modelling.

**Method:**

We developed a graph attention network model for amyloid and tau propagation with the following principles: brain network provide the network basis of pathological protein seeding; the pathology burden in a region can be predicted by its neighbouring regions; the likelihood of propagation between adjacent regions is determined by the transcriptomic profile in both regions. DTI data from HCP project was used to generate brain structural network for modelling. 1655 ^18^F‐Florbetapir PET (amyloid positive) and 522 ^18^F‐Flortaucipir (Tau positive) PET scans from in ADNI database were used to obtain amyloid and Tau pathology spatial distribution information. Micro‐array data from Allen brain atlas was used to represent the transcriptomic topology of healthy human brain (Figure 1). The data were then fed into the graph network model to learn the contribution of each gene to pathological protein propagation. Over representation analysis (ORA) was then performed to identify key pathways represented in the top (positive) and bottom (negative) 5% genes.

**Result:**

The models were able to predict regional pathology based on its neighbouring nodes (mean squared error <0.01 for amyloid and <0.1 for Tau scans). Many top weighted genes are known to be associated with AD, including APOE, B2M, HLA, etc. ORA indicated that synaptic transmission and calcium channel regulator pathways facilitated the inter‐regional propagation of tau pathology, while innate immune pathways were protective against Tau seeding (Figure 2). Innate immune pathways were also suggested to be protective against amyloid pathology propagation (Figure 3).

**Conclusion:**

We present a novel framework that enables integration of microscopic and macroscopic data in neurodegeneration. Our model suggested constitutional expression of innate immune molecules are protective against Alzheimer's pathologies’ propagation, and supports a protective role of neuroinflammation in early AD.